# Improving the application of quantitative fatty acid signature analysis in soil food webs: The effects of diet fat content

**DOI:** 10.1002/ece3.7894

**Published:** 2021-07-09

**Authors:** Jakob Kühn, Vanessa Henning, Liliane Ruess

**Affiliations:** ^1^ Institute of Biology Ecology Group Humboldt‐Universität zu Berlin Berlin Germany

**Keywords:** Collembola, lipids, quantitative fatty acid signature analysis, soil food webs

## Abstract

Quantitative fatty acid signature analysis (QFASA) as a biochemical tool to study the diet composition of predators is frequently used in marine ecology to infer trophic links in vertebrate consumers. However, the potential and challenges of this method in other ecosystems have only recently been studied. The application in soil ecosystems leads to hurdles not encountered in the marine, such as the low similarity of fatty acid signatures between resource and consumer. So far, diet estimation attempts have been semisuccessful, necessitating to adapt QFASA for use in soil food webs. Dietary fat content may play an important role, as it influences consumer metabolism, and thus calibration coefficients for fatty acid trophic transfer. A series of feeding trials with baker's yeast spiked with five different pure fatty acids at various concentrations was conducted with Collembola, and the changes in calibration coefficients were observed. From there, equations were gained through regression analysis and new sets of calibration coefficients were calculated. QFASA was applied on a range of basal resources and the results compared with previously defined calibration coefficients. Calibration coefficients changed with the proportion of fatty acids in the diet and differed between the three Collembolan species. The re‐estimation of diets showed an improvement of model performance by the new calibration coefficients and indicated several modes of fatty acid assimilation. These greatly influence the outcome of diet estimation, for example, algal and bacterial diets are likely underestimated due to high metabolic turnover rates. The application of QFASA in soil ecosystems remains challenging. The variation in calibration coefficients and the resulting decrease in estimation deviation indicate the merit of calculating calibration coefficients from consumer signatures through linear or exponential equations. Ideally, the method should, when extended to the entire fatty acid signature, allow correct determination of consumer diets in soil food webs.

## Graphical abstract

The establishment of QFASA in soil food webs remains challenging due to low overlap in signature between resource and consumer and highly variable metabolic responses, impacting calibration coefficients. A series of experimental trials with baker's yeast spiked with five different pure fatty acids at various fat levels was conducted with Collembola, and the changes in calibration coefficients were observed. Calibration coefficients generally changed with the proportion of fatty acid in the diet and were markedly different between the three Collembolan species.

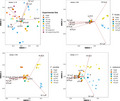

## 1 INTRODUCTION

A key aspect of soil ecological research is the investigation of energy flow in food webs via trophic links, that is, the resource–consumer relationships. In soil ecology, it is generally assumed that energy and matter are bound in discrete “channels” representing decomposer systems dominated by fungi, bacteria, or plants (Heijboer et al., 2017). The prominence of these channels is habitat‐specific, and thus, a precise understanding of carbon and energy flow through these pathways is necessary to gain insights into food web functioning. Modern biochemical tools such as stable isotope (Potapov et al., 2019; Twining et al., 2020) and fatty acid analysis (Ruess & Chamberlain, 2010; Traugott et al., 2013) have become the standard in discerning such trophic interactions in soil food webs.

In marine and freshwater ecology “quantitative fatty acid signature analysis” (QFASA; Iverson et al., 2004) is a widely applied method to infer the diet composition of vertebrate predators such as fish (Budge et al., 2012; Happel et al., 2016), sea birds (Williams & Buck, 2010), and mammals (Goetsch et al., 2018; Meynier et al., 2010). Briefly, QFASA functions by assuming a consumers’ fatty acid signature to be a linear mixture of its diets’ signatures modified by consumer physiology. For the latter, calibration coefficients (CCs) are used as weighting factors that account for the effects of consumer metabolism on lipid patterns. Only recently, QFASA has been explored in soil ecosystems: After establishment of a reference library for common basal soil resources (Kühn et al., 2019), a first set of CCs for Collembola was calculated. An estimation of mixed diets was performed using the QFASA model, albeit only partly successful by identification of the main food components but not delivering the correct proportions (Kühn et al., 2020).

As the QFASA algorithm bases its calculation on the proportion of dietary fatty acids incorporated into the consumers’ lipid signature, factors with impact on this proportion are a prime target to advance the model. While ideally the CCs of a fatty acid should be close to static, several studies have shown CCs vary strongly within the same consumer, when fed on different resources (Magnone et al., 2015; Rosen & Tollit, 2012) as well as with the fat content of the diet (Budge et al., 2020). Similarly, the study focusing on QFASA in soil ecosystems observed variation in CCs between diets (Kühn et al., 2020), suggesting the fat content to be particularly important due to the generally low quality of soil food web resources. There is need to assess the underlying relationships that influence CCs and incorporate these into the QFASA model. Moreover, Collembola showed species‐specific differences (Kühn et al., 2020), likely ecological adaptations to the habitat requirements. Generalistic feeding is common among soil decomposers (Scheu, 2002), although there certainly are feeding preferences of faunal decomposers (Brückner et al., 2018). Particularly in deeper soil horizons, generalistic feeding allows utilization of a wider food range and, in turn, selection of resource‐specific fatty acids (Endlweber et al., 2009).

To get a mechanistic understanding of the trophic shift of fatty acids between soil decomposers and their resources, laboratory feeding trials with Collembola were preformed using yeast diet spiked with six different pure fatty acids, offered solely or in combination at three different fat levels. Thereby, a formulated diet was constructed with a defined fatty acid signal, allowing for quantitative follow‐up in the consumer. Two classes of trophic marker fatty acids were tested: absolute markers that the consumer is unable to synthesize itself and relative markers that are constitutes of its metabolic pathways but increase in proportion if certain resources are fed upon (Ruess & Chamberlain, 2010). The aim of this study is to assess the impact of varying dietary fatty acid proportions on the CCs and diet estimation in mesofauna consumers for the enhancement of the application of the QFASA model in soil food webs. Further, the potential of calculating CCs from consumer signatures directly, which would capture the relationship between dietary signature, CC, and consumer signature, is assessed. These revised CCs are tested, using the lipid pattern of a prior experiment with Collembola feeding on natural soil resources (Kühn et al., 2020) to reveal the improvement in model performance.

## 2 MATERIAL AND METHODS

### 2.1 Cultivation of Collembola

The experiment focused on three species of Collembola representing three life strategies: the epedaphic *Lepidocyrtus violaceus* (Fourcroy, 1785), the hemiedaphic *Folsomia candida* (Willem, 1902), and the euedaphic *Protaphorura fimata* (Gisin, 1952). Prior to the experiments, the Collembola were cultivated in plastic microcosms, filled partially with a layer of a mixture of plaster, water, and activated charcoal in a ratio of 8:7:1. During the cultivation, Collembola were kept in dark conditions at 15°C and fed with baker's yeast (*Saccharomyces cerivisae*) for a minimum of 4 weeks to ensure a homogenous fatty acid signature before the start of the experiment.

### 2.2 Experimental setup

In the experiments, Collembola were kept at 15°C in the dark and fed on diets of pure baker's yeast (*Saccharomyces cerivisae*) mixed with varying concentrations of specific marker fatty acids. The relative markers oleic acid (18:1ω9) for plant and linoleic acid (18:2ω6) were used for fungal feeding as well as the absolute markers 13‐methyl pentadecanoic acid (a15:0), lactobacillic acid (cy19:0), 11‐hexaenoic acid (16:1ω5), and 7,10,13‐hexadecatrienoic acid (16:3ω3), representing absolute markers for feeding gram‐positive bacteria, gram‐negative bacteria, arbuscular mycorrhiza fungi, and green algae, respectively (Buse et al., 2013; Ngosong et al., 2014; Ruess et al., 2005).

Diets were formulated by adding 0.25 g of powdered baker's yeast into a liquid mixture of 0.5 ml double‐distilled water, 40 µl acetone, and 13.2 mg (amounting to 5% of added fatty acid), 27.8 mg (amounting to 10%), or 44.1 mg (amounting to 15%) of liquid or crystalline pure nonmethylated fatty acid (adapted from Malcicka et al., 2017). Diets were fed in either single diet setups with varying (5%, 10%, or 15%) amounts of added fatty acid, or mixed diet setups with multiple fatty acids added in identical concentrations of 5% per fatty acid. Mixtures of 18:1ω9 and a15:0 as well as a mix of 18:1ω9, a15:0, and cy19:0 were fed to investigate the effects of feeding both relative and absolute markers. Furthermore, trials were run for either 3 or 6 days to infer if the fatty acid‐specific incorporation and metabolization rates have a potential impact on the CCs. An overview of all the trials is given in Table S1.

### 2.3 Extraction and identification of FAMEs

After the feeding trial, Collembola cultures were frozen and stored in 15‐ml reaction tubes at −20°C until fatty acid extraction. Fatty acid signatures of all experimental treatments were extracted via a four‐step acidic methanolysis procedure (MIDI Inc., Newark, Del), consisting of saponification, methylation, extraction, and washing steps, with the addition of nonadeconaoic acid (19:0) as an internal standard. In the resulting total lipid fatty acid (TLFA) fraction, which incorporates both dietary effects visible in the NLFA fraction and consumer metabolic effects in the PLFA fraction, fatty acid methyl esters (FAME) were identified via gas chromatography coupled to a flame ionization detector (GC‐FID, Agilent 7890 A). After identification, fatty acid identification was validated by structure analysis using mass spectrometry (GC‐MS, Agilent 7890 A, Agilent 7000 Triplequadrupole). For method details, see Kühn et al. (2019).

### 2.4 Data handling and statistical analysis

The TLFA signatures of the diet and collembolan consumers were transformed from FID response values into biomass concentrations using the internal standard as reference, expressed as µmol fatty acid g^−1^ fresh weight for further analysis. Nonmetric dimensional scaling (nMDS) analysis was conducted on the biomass data to visualize groupings in the signatures and the effect of increased fatty acid abundance in the diet on consumer tissue, followed by PERMANOVA and PERMDISP analyses to test for statistical differences in the ordination. Multiple analyses of variance (ANOVA) with post hoc Tukey's honest significant difference tests were carried out to infer whether the fatty acid concentrations between the feeding trials were significantly different.

For further analysis related to the QFASA algorithm, biomass data were transformed to fatty acid proportion signatures, which form the basis of QFASA estimation (Iverson et al., 2004). A consumers’ diet is thereby estimated by assigning a mixture of diet signatures that minimizes the multivariate distance (i.e., Aitchison distance) to the consumer signature. Diet signatures are referenced from a lipid library, that is, a dataset of 229 fatty acid signatures of soil resources (Kühn et al., 2019). Consumer signatures are transformed from the “consumer space” to the “resource space” via the calibration coefficient set, which account for consumer metabolic changes are unique to the consumer species (Iverson et al., 2004).

To assign the effect strength of the experimental fatty acid supplementation, the concentrations of the marker fatty acids in the consumer signature were compared to a baseline of Collembola consumers fed on pure yeast. The differences in marker fatty acid concentrations (ΔFA) were calculated by subtracting the concentration of the marker fatty acid in the lipid pattern of the yeast‐fed animals from the concentration of the marker fatty acid in the signatures of the Collembola in the experimental treatments.

CCs were calculated as the division quotient of the fatty acid proportion in the consumer and the diet, using all diet and consumer samples (i.e., 9 CCs per fatty acid per collembolan species for every experimental treatment). The relationship between the fatty acid proportion in the consumer and the respective CC was investigated through linear regression, plotting the proportion of the experimentally enriched fatty acid in the consumer with the calibration coefficient array for that fatty acid, including all three levels of the formulated diet (i.e., 27 responses per plot).

A second regression model was conducted, comprising data from the present experiment as well as data of Collembola fed on natural soil resources (Kühn et al., 2020). Both linear and exponential regression were conducted, where applicable. The resulting regression functions were used to calculate new CC sets for the collembolan consumers fed on mixed natural soil resources (Kühn et al., 2020) on a per‐trial basis, and new QFASA estimations for Collembola diets consuming mixed natural resources were performed. These results were compared to the results presented in Kühn et al. (2020), that is, the average deviation of the estimated diet from the diet composition that was actually fed. These deviations were calculated by subtracting the estimated proportion of a diet component from the “true” proportion fed in the mixed diet trial (e.g., an estimation of 20% from a diet that constituted 50% of the diet results in 30% deviation) and summing up the amounts over all diet components. The overall deviation given for a species is the average over all summed deviations.

All statistical analyses and graphical representations were realized in R version 4.0.2. The nonmetric dimensional scaling analysis was conducted using the vegan package (Oksanen et al., 2018). The diet estimation was conducted using the qfasar package (Bromaghin, 2017a).

## 3 RESULTS

### 3.1 Lipid pattern

The feeding trials resulted in a dataset containing 432 entries from 38 setups comprising 31 fatty acids ranging from 11 to 22 carbon atom chain length, containing both 3‐day and 6‐day duration trials (Table S2). Besides the marker fatty acids used as experimental treatments, the lipid pattern of yeast and Collembola comprised up to 8 saturated and 16 unsaturated fatty acids, as well as 5 methyl‐branched (i.e., iso‐ and anteiso‐branched) and 2 cyclic fatty acids, ranging from a carbon chain length between 11 and 20 (see Table S2).

**FIGURE 1:**
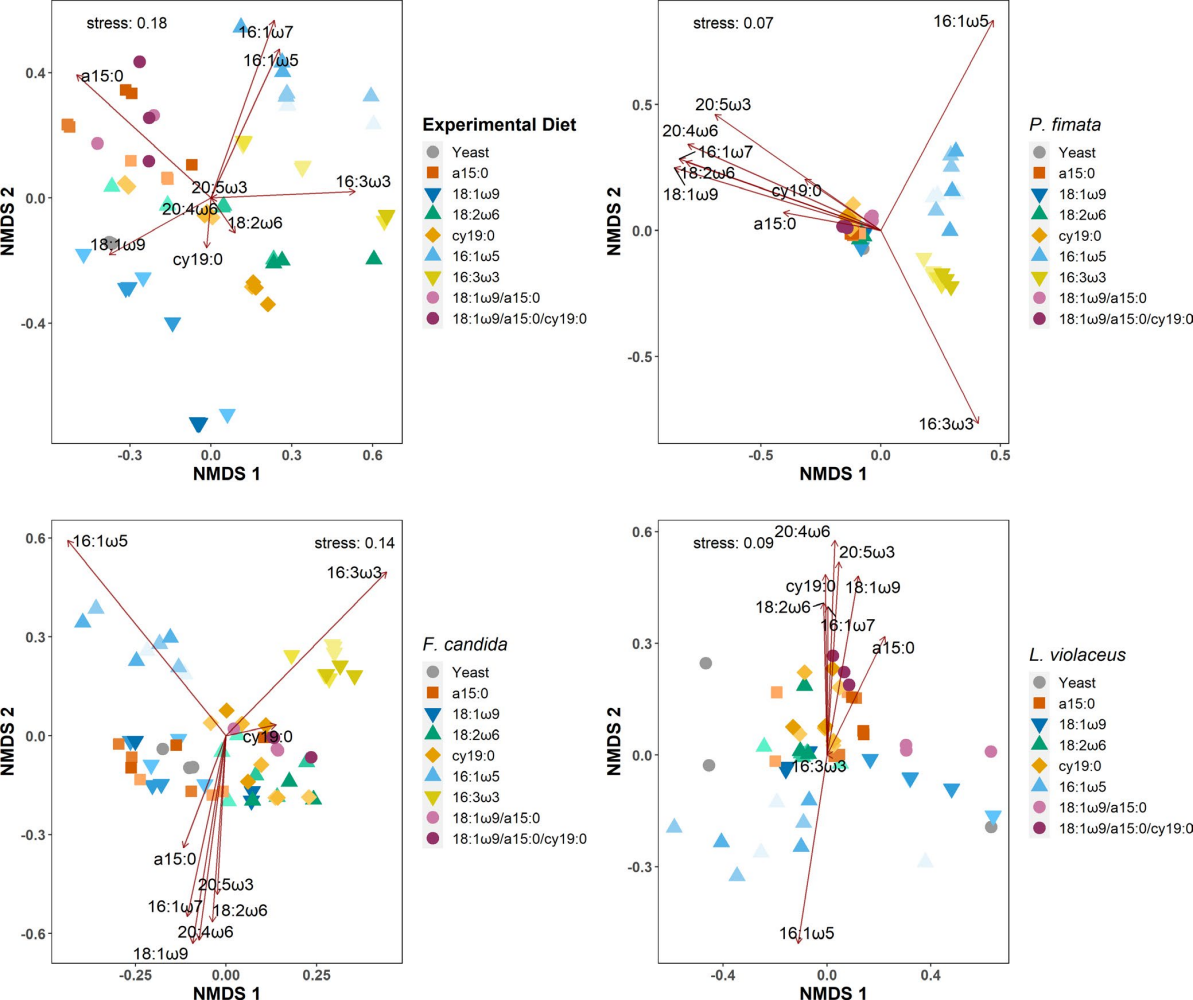
Nonmetric multidimensional scaling (nMDS) analysis of the fatty acid pattern of the experimental diets (upper left) and of the three Collembola consumers *Protaphorura fimata*, *Folsomia candida,* and *Lepidocyrtus violaceus* after 3 days of feeding. Shown are pure yeast and the designed diets containing additionally 5% (bright color), 10% (intermediate shade), and 15% (dark color) of the fatty acid 18:1ω9, 18:2ω6,9, a15:0, cy19:0, 16:1ω5, or 16:3ω3,6,9 as well as combinations with two and three different fatty acids

**FIGURE 2:**
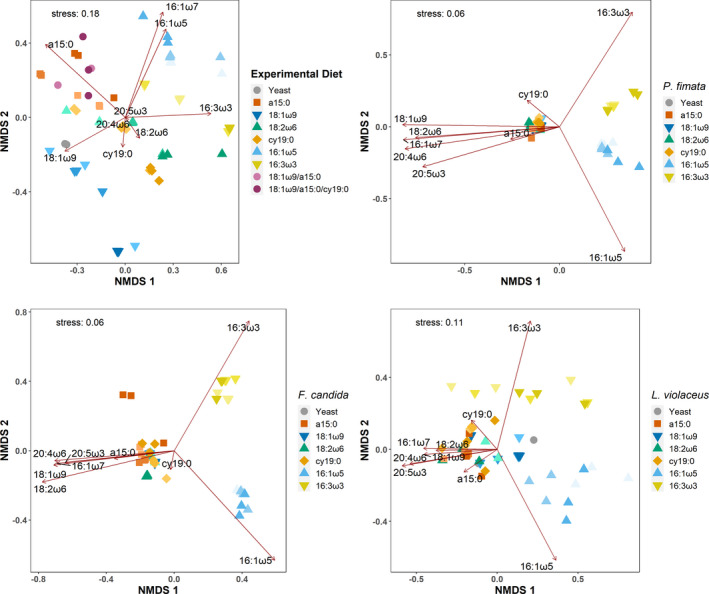
Nonmetric multidimensional scaling (nMDS) analysis of the fatty acid pattern of the experimental diets (upper left) and of the three Collembola consumers *Protaphorura fimata*, *Folsomia candida,* and *Lepidocyrtus violaceus* after 6 days of feeding. Shown are pure yeast and the designed diets containing additionally 5% (bright color), 10% (intermediate shade), and 15% (dark color) of the fatty acid 18:1ω9, 18:2ω6,9, a15:0, cy19:0, 16:1ω5, or 16:3ω3,6,9 as well as combinations with two and three different fatty acids

The nMDS for the lipid pattern of the diet as well as the Collembola exhibited a general trend toward structuring along the fat content gradient, with higher concentrations of supplemented marker fatty acid leading to higher concentrations in the fatty acid signature. This was most distinct in the diet (Figures 1 and 2). The nMDS with the experimental diets clearly shows the artificial nature, with the spiked diets spreading out from the pure yeast in the center in a star‐like pattern reflecting the increased fat content (light to dark color). PERMANOVA and PERMDISP of the ordination data revealed strong statistical differences between the groups and their dispersal patterns (PERMANOVA: pseudo‐*F*_20_ = 234.04, *p* = .02; PERMDISP: *F*_21_ = 3.13, *p* < .001). In Collembola, this picture was much less pronounced, with 3‐day feeding trials (Figure 1) only pointing to a structuring in *L. violaceus* and a differentiation in the 16 carbon chain length fatty acids in *F. candida,* but no clear trend in *P. fimata*. In the 6‐day feeding trials, the separation along the fat content was more apparent, yet with an overall low distance (Figure 2). PERMANOVA and PERMDISP only showed statistically significant differences in *P. fimata* for both 3 days (PERMANOVA: pseudo‐*F*_20_ = 313,818, *p* < .001, PERMDISP: *F*_21_ = 3.93, *p* < .001) and 6 days (PERMANOVA: pseudo‐*F*_20_ = 84,529, *p* < .001, PERMDISP: *F*_21_ = 4.94, *p* < .001).

### 3.2 Trophic transfer of fatty acids

An ANOVA of the marker fatty acid biomass data indicated fatty acid‐ and species‐specific differences (Table 1). Each of the diets in the experiment differed significantly from the base yeast diet, and in 18:2ω6, a15:0, 16:1ω5, and 16:3ω3, Tukey's HSD revealed significant differences between the different fatty acid concentrations. In Collembola, 9 out of 18 trials showed significant differences after a duration of 3 days and 15 out of 18 trials in the 6‐day experiments. 18:1ω9 did not significantly differ for any species in the 3‐day experiments, and no significant differences were observed in *F. candida* for the 6‐day experiment as well. *F*. *candida* generally displayed the weakest response to the formulated diets, only showing significant differences in 16:3ω3 at both time points, in the 3‐day experiment for 18:2ω6 and in the 6‐day experiments including for a15:0 and 16:1ω5. In *L. violaceus*, there were no significant differences observed in the 3‐day experiments featuring a15:0 and 16:1ω5.

**TABLE 1:** Average biomass [µmol/g fresh weight] of marker fatty acids in the signatures of pure yeast diet and the experimental trials as well as signatures of the three Collembola consumers *Protaphorura fimata*, *Folsomia candida,* and *Lepidocyrtus violaceus* in both 3‐ and 6‐day feeding experiments

Fatty acid	Yeast	3 days			ANOVA		Yeast	6 days			ANOVA	
		5%	10%	15%	*F*	*p*		5%	10%	15%	*F*	*p*
18:1ω9												
Diet	b	45.69ab	57.11a	39.39ab	5.29	.027						
*P. fimata*		347.55	262.53	300.85	2.31	.15	ab	264.07b	319.47ab	424.61a	6.19	.017
*F. candida*		126.45	117.70	120.85	0.21	.89		127.10	155.10	214.581	38.50	.27
*L. violaceus*		55.47	73.20	50.33	3.36	.07	b	65.70b	69.68ab	94.23a	10.09	.004
18:2ω6,9												
Diet	ac	41.26a	113.33b	43.66a	18.34	<.001						
*P. fimata*	b	66.35b	90.85ab	107.18a	5.80	.021	b	62.26b	105.76b	170.22a	24.27	<.001
*F. candida*	b	21.48ab	27.44ab	36.58a	4.53	.039		17.88	32.21	33.32	3.08	.09
*L. violaceus*	b	27.83ab	40.53a	42.73a	9.88	.004	c	97.19b	57.85a	50.91a	25.93	<.001
a15:0												
Diet	c	22.155ac	98.60b	49.33a	22.01	<.001						
*P. fimata*	b	16.30b	50.84a	66.54a	20.05	<.001	b	22.08b	57.20a	77.71a	20.39	<.001
*F. candida*		8.91	9.95	3.66	2.83	.11	c	9.55c	22.11b	4.19a	70.97	<.001
*L. violaceus*		6.81	18.59	16.02	3.94	.05	b	7.21b	22.70a	17.37a	29.21	<.001
cy19:0												
Diet	b	42.83a	69.21a	46.72a	18.86	<.001						
*P. fimata*	c	5.83bc	15.63b	28.06a	21.20	<.001	c	12.25b	23.83b	35.83a	49.22	<.001
*F. candida*		0.91	0.92	7.45	2.99	.10		0.53	0.00	0.00	1.00	.44
*L. violaceus*	d	4.62c	9.38b	13.87a	92.87	<.001	d	6.91c	12.17b	17.65a	46.13	<.001
16:1ω5												
Diet	b	36.36b	82.54ab	140.25a	9.32	.005						
*P. fimata*	b	4.22ab	7.72a	7.45a	6.93	.013	b	6.13ab	6.11ab	12.91a	8.59	.007
*F. candida*		4.62	8.39	10.18	3.01	.09	b	12.60ab	12.50ab	15.61a	4.83	.03
*L. violaceus*		1.43	1.99	8.52	3.49	.07	b	1.13ab	2.89a	3.26a	6.19	.018
16:3ω3,6,9												
Diet	c	64.01bc	80.75b	176.92a	26.48	<.001						
*P. fimata*	b	4.50bc	10.98a	7.76ac	11.90	.003	bc	3.67a	2.51ac	2.42ac	6.51	.015
*F. candida*	c	5.21b	1.75a	0.99ac	63.39	<.001	c	4.90b	4.22b	1.96a	27.42	<.001
*L. violaceus*	–	–	–	–	–	–	b	18.16ab	3.41a	6.51a	23.23	<.001

Note

Given are ANOVA *F* and *p* values and Tukey's HSD (*p* < .05). Letters indicate significant differences between the experiments within one species.

**FIGURE 3:**
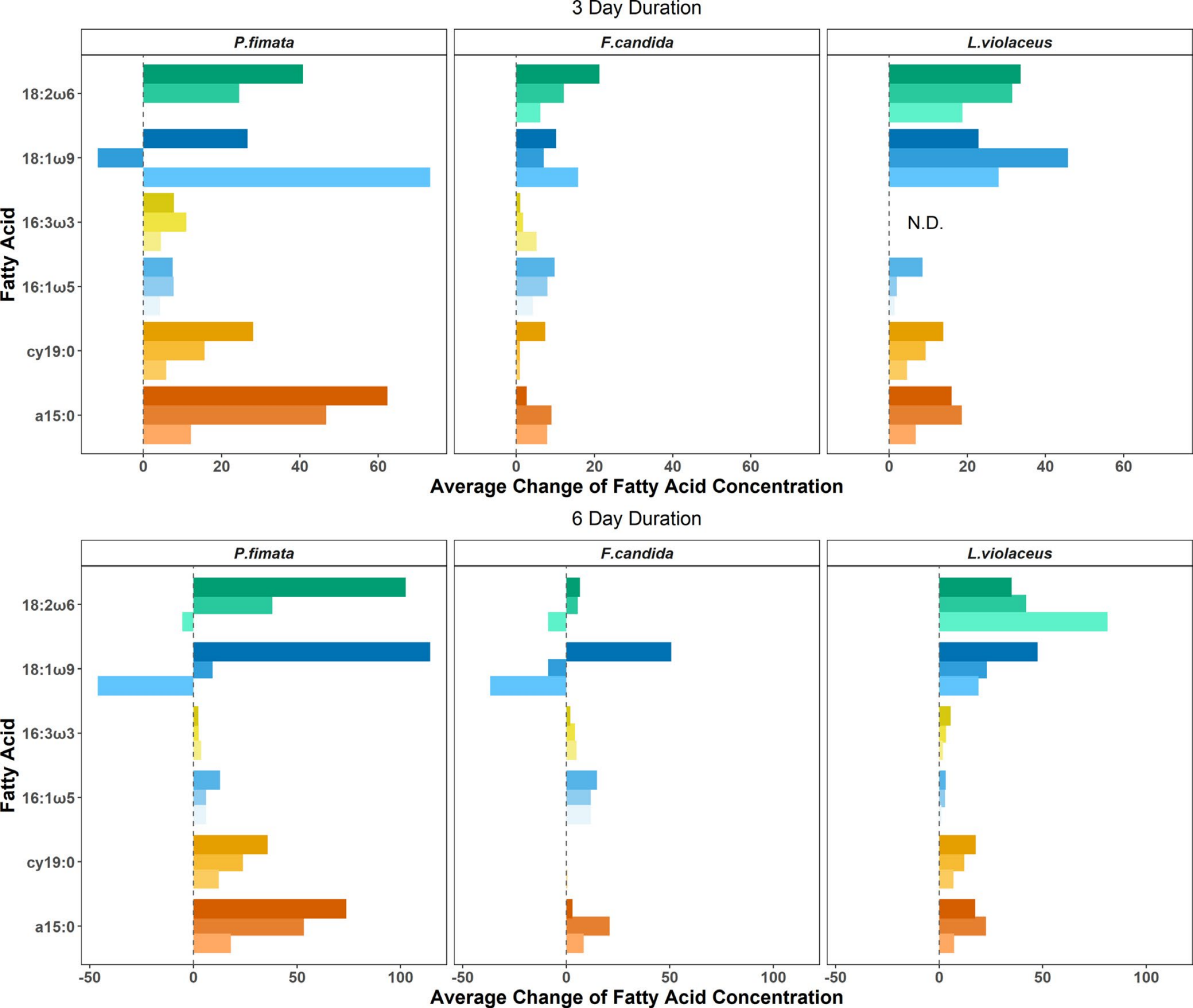
Incorporation of fatty acids (FA) supplemented in the designed experimental diets relative to a baseline signature (ΔFA). Shown are the changes in the fat concentrations in consumers when fed on diets containing 5% (bright color), 10% (intermediate shade), and 15% (dark color) of the fatty acid 18:1ω9, 18:2ω6,9, a15:0, cy19:0, 16:1ω5, or 16:3ω3,6,9 in relation to a pure yeast diet

Comparing the level of incorporation relative to a pure yeast baseline (ΔFA) revealed a species‐specific and fatty acid‐specific response toward the experimental diets with various levels of supplemented fatty acids (Figure 3). The relative markers 18:1ω9 and 18:2ω6 showed very variable patterns, particularly in *P. fimata* and *F. candida*, while in *L. violaceus* the assimilation of fatty acids was reduced with increasing fatty acid concentration in the diet in the 6‐day experiment for 18:2ω6 and the 3‐day experiment for 18:1ω9. Meanwhile, increasing concentrations of the absolute markers 16:1ω5, a15:0, and cy19:0 resulted in an enhanced assimilation of the respective fatty acid in most feeding trials in both 3‐ and 6‐day experiments. Lastly, 16:3ω3 showed only minor assimilation regardless of concentration in the diet.

When fed on mixed diets, the level of incorporation of the different fatty acids into consumer tissue showed species‐ and fatty acid‐specific responses to the mixed diets (Table 2). In the relative marker 18:1ω9, the abundance of the marker was only significantly lower in *F. candida*, whereas *L. violaceus* showed only significantly lower levels of 18:1ω9 in the triple diet mix (18:1ω9, a15:0 and cy19:0) compared to the level of 18:1ω9 in the single cy19:0 diet. *P. fimata* showed no significant differences in the abundances of 18:1ω9. In contrast, the biomass of the absolute marker cy19:0 increased in Collembola consumers feeding a mixed diet in comparison to the respective single fatty acid diet setups in all three species. The absolute marker a15:0 in the mixed diet was observed in intermediate levels in *F. candida* in comparison to the single diet, *L. violaceus* showed comparable levels of marker fatty acids in both single and mixed diet, while in *P. fimata,* its amounts were higher in the mixed diet than in the respective single diet, but not significantly so.

**TABLE 2:** Average biomass [µmol/g fresh weight] of the marker fatty acid in the three Collembola consumers *Protaphorura fimata*, *Folsomia candida,* and *Lepidocyrtus violaceus*

Species	Diet mix	Single diet component				ANOVA	
						*F*	*p*
		Combined diet	18:1ω9	a15:0	cy19:0		
*L. violaceus*	**18:1ω9**/a15:0	64.51	55.47	51.35	–	1.07	.4
	**18:1ω9**/a15:0/cy19:0	52.11b	55.47ab	51.35ab	89.79a	5.66	<.02*
*F. candida*	**18:1ω9**/a15:0	104.94b	126.45b	216.82a	–	31.61	<.001***
	**18:1ω9**/a15:0/cy19:0	123.12b	126.45b	216.82a	198.92a	46.15	<.001***
*P. fimata*	**18:1ω9**/a15:0	320.57	347.66	423.16	–	2.27	.18
	**18:1ω9**/a15:0/cy19:0	319.02	347.66	423.16	416.08	3.76	.06
*L. violaceus*	18:1ω9/**a15:0**	18.43	0	6.81	–	2.61	.15
	18:1ω9/**a15:0**/cy19:0	10.25a	0b	6.81a	0b	23.31	<.001***
*F. candida*	18:1ω9/**a15:0**	4.48ab	1.63b	8.91a	–	4.87	.06
	18:1ω9/**a15:0**/cy19:0	5.77ab	1.63b	8.91a	0.93b	11.71	.002**
*P. fimata*	18:1ω9/**a15:0**	28.40a	5.88b	16.30a	–	12.79	.007**
	18:1ω9/**a15:0**/cy19:0	23.51a	5.88b	16.30ac	7.92bc	17.44	<.001***
*L. violaceus*	18:1ω9/a15:0/**cy19:0**	10.07a	0c	0c	6.91b	27.89	<.001***
*F. candida*	18:1ω9/a15:0/**cy19:0**	4.55a	0b	0b	0.91b	2.82	<.001***
*P. fimata*	18:1ω9/a15:0/**cy19:0**	18.88a	0a	0a	5.83b	25.36	<.001***

Note

Presented are the proportions of the fatty acid marked in bold in the combined diet setup in comparison to their respective single diet setups. Given are ANOVA *F* and *p* values and Tukey's HSD (*p* < .05). Significances are given as *, **, *** with *p* < .05, .01, and <.001. Letters indicate significant differences between the experiments within one species.

### 3.3 Revision of calibration coefficients

**FIGURE 4:**
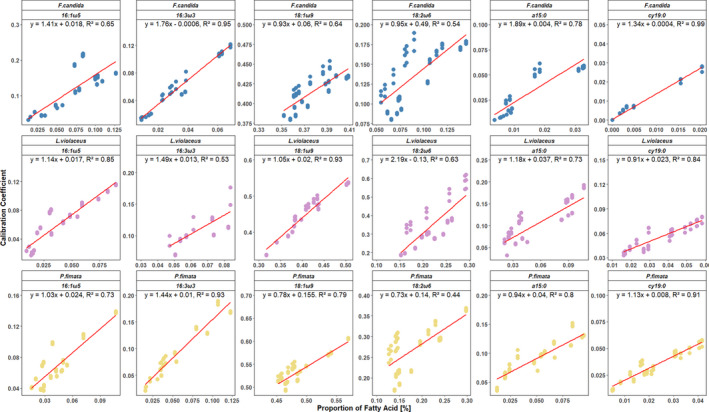
Linear regression of fatty acid proportion [%] in the consumer signature and their respective calibration coefficients. Shown are the regressions for all fatty acids supplemented in the design diets—18:1ω9, 18:2ω6,9, a15:0, cy19:0, 16:1ω5, and 16:3ω3,6,9 fed to the Collembola *Protaphorura fimata, Folsomia candida,* and *Lepidocyrtus violaceus*

**FIGURE 5:**
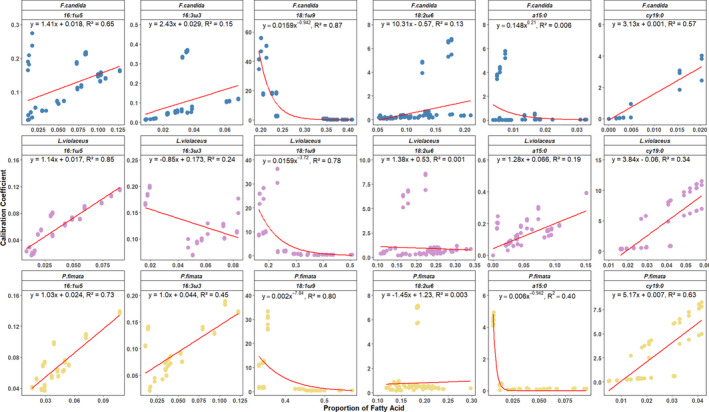
Regression of fatty acid proportion [%] in the consumer signature and their respective calibration coefficients for a combined dataset of the experiment presented in this study and feeding data of a prior, related publication (Kühn et al., 2020). Shown are the regressions for all fatty acids supplemented in the design diets—18:1ω9, 18:2ω6,9, a15:0, cy19:0, 16:1ω5, and 16:3ω3,6,9 fed to the Collembola *Protaphorura fimata, Folsomia candida,* and *Lepidocyrtus violaceus*

Based on the results above, the CCs of the single diet experiments were plotted against the proportion of the supplemented fatty acid in the consumer signature to visualize the change in CC in relation to consumer FA proportion (Figure 4). CCs and relative fatty acid proportion were generally positively correlated, although the slope was very low. R² ranged from 0.43 in 18:2ω6—*P. fimata* to 0.93 in 18:1ω9—*L*. *violaceus*. However, when paired with results from the feeding experiments on natural resources (Kühn et al., 2020), data formed a negative exponential curve in 18:1ω9, with the design experiment data presented here showing the lowest values, whereas in 18:2ω6 results were almost constant (Figure 5).

The resulting linear equations formed the basis to the formulation of new CCs accounting for the consumer's fatty acid proportion. These were then applied in a new signature analysis using the lipid pattern of Collembola feeding on the common soil resources algae, bacteria, fungi, and green plants leaf and root tissue (Kühn et al., 2020). The results of the estimation of the revised CCs are presented in Table S3. Generally, the overestimation of root diet by the old CCs went down, while the assessment of algae and fungi as resource went up. However, the latter partly overstated the consumption of fungal diet by Collembola. The average deviation, expressed as the mean of the summed deviations from the presented diet mix, was improved in all three species (*Folsomia candida*: 84% ± 16, down from a deviation of 92% ± 16; *Protaphorura fimata*: 75% ± 14, down from 90% ± 15; *Lepidocyrtus violaceus*: 93% ± 16, down from 113% ± 18), although these decreases were not significant. Overall, the diet estimation in Collembola became better in quality for 19 out of 30 cases.

## 4 DISCUSSION

### 4.1 Effects of Collembola metabolism on CCs

Using defined, formulated diet showed that CCs in Collembola did change with dietary fatty acid proportions in both the 3‐ and 6‐day feeding experiments. The assimilation of the supplemented fatty acids increased in Collembola lipids, yet this was much more pronounced after feeding for a 6‐day period. This indicates that QFASA in general reflects long‐time dietary differences, not short‐time diet switches. The incorporation rate of fatty acids in Collembola tissue shows notable differences between common unsaturated fatty acids like 18:1ω9 and 18:2ω6, which take very little time to accumulate, and methyl‐branched fatty acids, which take much longer (Chamberlain et al., 2005). Bacterial iso‐ and anteiso fatty acids showed no isotopic shift in δ^13^C values between diet and consumer over 14 days, suggesting no or only minor alteration by Collembola lipid metabolism (Chamberlain et al., 2006). Presumably, after a diet‐switch, it takes a few days to allow the fatty acid signature to notably change, in particular in bacteria‐derived fatty acids.

The observed changes in CCs (Figure 5) can be placed into three categories: Two of these are characterized by CC above 1, indicating fatty acids that are either preferentially taken up or synthesized de novo by Collembola. In all three species, 18:1ω9, as well as a15:0 in *P*. *fimata* and *F*. *candida*, exhibited very high CCs at low proportions in the diet, which rapidly fell toward a low CC plateau (below 1). Of them, 18:1ω9 is one of the main fatty acids in insects and serves a variety of functions such as maintaining homeostasis (e.g., Michaud & Denlinger, 2006) and acts as a precursor to linoleic acid (Malcicka et al., 2017). 18:1ω9 is therefore highly regulated by physiological processes in the consumer, making synthesis by the consumer likely. This is in line with the low variation in the consumer signatures in contrast to the diet. Likewise, methyl‐branched fatty acids, which are widespread in bacterial membrane lipids (Zelles, 1999), are supposed to be a precursor for various pheromones and hormones in insects (Juárez et al., 1992) and thus are potentially preferentially assimilated. However, Haubert et al. (2006) report that these branched‐chain molecules are not biosynthesized by Collembola. This suggests that the accumulation of a15:0 is the result of a lack in enzymatic capacity for processing this particular fatty acid, leading to storage and increased presence in the consumer lipids, at least in *F. candida*.

In contrast to 18:1ω9, and a15:0, the fatty acid 18:2ω6,9 was assimilated into Collembola tissue at a constant rate, with a static CC. Linoleic acid is an important precursor in a variety of metabolic functions (Brandstetter & Ruther, 2016; Meyer et al., 2003) and the synthesis of arachidonic acid (Menzel et al., 2017), important for insect immune responses. Therefore, larger pools of linoleic acid in the diet allow larger deposition rather than utilization in the consumer, resulting in constant CCs across dietary fat content.

In comparable studies on mites, the assimilation rate of fatty acids from both low fat and high fat resources correlated with the amount in the consumer. Further, some fatty acids were highly linked to the diet (e.g., 18:1ω9), whereas others such as 18:2ω6 were not (Brückner et al., 2017), showing both similar as well as contrasting patterns to Collembola in this study. On the other hand, studies on ants show 18:2ω6 to increase in consumer tissue with an increasing amount in resource tissue (Rosumek et al., 2017), similar to the results presented here.

The third category, comprising the fatty acids cy19:0, 16:1ω5, and 16:3ω3, all exhibited CC values far below 1. This indicates that these FAs are preferentially utilized, that is, subjected to catabolism or structural changes (elongation, desaturation) before deposition. However, the CCs increased with an increasing proportion of these fatty acids in the diet. These fatty acids constitute absolute trophic markers for bacteria, arbuscular mycorrhiza, and green algae, respectively (Buse et al., 2013; Ngosong et al., 2014; Ruess et al., 2005). Presumably, they are directly metabolized, with only a high proportion of the respective fatty acid in the diet leading to increased accumulation. This can cause an underrepresentation of bacterial or algal diets, particularly if they constitute rare components of the Collembola diet. Although leading to high Collembola fitness, particularly algae are often neglected as a diet in comparison to fungi or plants (Buse et al., 2013), which are presumed to be the dominant resources for the majority of Collembola (Ponge, 2000).

Notably, the CC sets gained here are markedly different from the sets gained from Collembola fed on natural diets (Kühn et al., 2020), raising the question whether both CC sets can be combined to form the equations discussed above. While the proportions of the supplemented marker fatty acids are in excess of what is found in comparable natural diets, the chosen marker acids constitute the major fatty acids for the respective resource (see Kühn et al., 2019) and thus are representative model molecules to reflect the natural diet. However, the results of this experiment indicate a saturation at all fat contents investigated. Therefore, the effect of assimilation was not completely captured, as data for an “intermediate” level of fatty acids are missing. Future experiments featuring supplemented yeast spiked with lower levels of marker fatty acids (e.g., 0.05%, 0.1%, and 0.5%) should fit this data gap. Similar effects were observed in marine QFASA research (Budge et al., 2020), where the diets in many feeding experiments already exceeded the fat levels representative for natural food. Further, as dietary resources in soil are typically low in fat, the rate of de novo fatty acid synthesis is high (Fernando‐Warnakulasuriya et al., 1988; Stanley‐Samuelson et al., 1988), leading to soil consumer metabolism with very variable CCs. This poses an additional challenge for the application of QFASA in terrestrial compared to marine food webs. Similarly, the small number of fatty acids in the signature of soil consumers and resources in comparison to the marine organisms for which QFASA was originally developed might amplify the bias introduced through variable assimilation rate, particularly when fat content is low and certain marker fatty acids cannot be traced to the consumer.

### 4.2 Application of revised CCs

To gauge the effect of the CCs gained in this new dataset on formulated diets, they were tested in estimating natural diets, thereby comparing the effectiveness of diet quantification by the QFASA model in soil ecosystems. The new estimation of mixed diets first presented in Kühn et al. (2020) did somewhat improve, reducing the deviation from the diet composition, particularly decreasing the overestimation of root diet. However, a correct estimation, that is, of all diet components with only 5% deviation, was still only achieved in one case. Comparing the CCs derived by the two studies, the largest value change appeared in 18:1ω9, where the combination of both datasets revealed an exponential relationship between fat content and CC. In contrast, other fatty acids in the design diet experiments resulted in far smaller changes in the CC.

Another potential problem in this evaluation of CC changes with fatty acid content is the constant sum constraint inherent in compositional data (Brückner & Heethoff, 2017), as changes in one component always affects the rest of the signature as well, and target FAs can be influenced by changing nontarget FA as well. Transforming the data to no longer be constrained might be a solution for this problem; however, so far, QFASA requires data to sum to 1 (Bromaghin et al., 2016) and therefore extensive changes to the model would have to be made.

Nonetheless, the results indicate that CCs calculated from regression data bear potential in correctly estimating the diets of soil consumers. Similar was shown in an experiment where the calculation of CCs was conducted simultaneously to the diet estimation from the same consumer signatures (Bromaghin, 2017b). A follow‐up study therefore should aim to identify the fatty acids with the highest variation due to metabolic processes in the consumer and establish linear or exponential equations depending on the preferential pathways in consumer metabolism. Generally, highly variable CCs reflect the strong metabolic regulation of certain fatty acids by the consumer and such control should result in similar exponential results as presented here. In contrast, purely dietary routed fatty acids are expected to give results similar to the absolute markers in this study.

Moreover, formulating yeast diets with comparable fat level additions as used in the present study as well as lower levels to better capture the changes at mid‐level ranges is advisable. Further, for the applicability of QFASA in soil systems on the lowest trophic level, the low diversity of fatty acids in consumers compared to basal resources has to be considered. At that food web level, this leads to low estimation power and increases the bias introduced by the constraints of compositional data. Changing the data from compositions to “open” data in future alterations to the QFAS model would potentially remove these biases that, while not apparent in marine ecology, are skewing results in soil systems due to the smaller footprint of fatty acid signatures. In sum, the presented methodology, when expanded to include every fatty acid in a Collembola lipid signature and taking into account the biases of compositional data, shows promise to establish the QFASA algorithm as a tool in soil food web ecology.

## CONFLICT OF INTEREST

No conflict of interest is declared.

## AUTHOR CONTRIBUTIONS

**Jakob Kühn:** Conceptualization (equal); Data curation (lead); Formal analysis (lead); Investigation (lead); Methodology (lead); Visualization (lead); Writing‐original draft (lead). **Vanessa Henning:** Data curation (supporting); Investigation (supporting); Methodology (supporting). **Liliane Ruess:** Conceptualization (equal); Funding acquisition (lead); Project administration (lead); Supervision (lead); Validation (supporting); Writing‐original draft (supporting); Writing‐review & editing (supporting).

## Supporting information

Table S1

Table S2

Table S3

## Data Availability

The minimal raw dataset is available at the public repository figshare (https://doi.org/10.6084/m9.figshare.14077022).

## References

[ece37894-bib-0001] Brandstetter, B., & Ruther, J. (2016). An insect with a delta‐12 desaturase, the jewel wasp *Nasonia* *vitripennis*, benefits from nutritional supply with linoleic acid. The Science of Nature, 103, 1–4. 10.1007/s00114-016-1365-0 27116611

[ece37894-bib-0002] Bromaghin, J. F. (2017a). qfasar: Quantitative fatty acid signature analysis in R. R package version 1.2.0. https://CRAN.R‐project.org/package=qfasar

[ece37894-bib-0003] Bromaghin, J. F. (2017b). Simultaneous estimation of diet composition and calibration coefficients with fatty acid signature data. Ecology and Evolution, 7, 6103–6113. 10.1002/ece3.3179 28861216 PMC5574754

[ece37894-bib-0004] Bromaghin, J. F., Budge, S. M., & Thiemann, G. W. (2016). Should fatty acid signature proportions sum to 1 for diet estimation? Ecological Research, 31, 597–606. 10.1007/s11284-016-1357-8

[ece37894-bib-0005] Brückner, A., & Heethoff, M. (2017). A chemo‐ecologists’ practical guide to compositional data analysis. Chemoecology, 27, 33–46. 10.1007/s00049-016-0227-8

[ece37894-bib-0006] Brückner, A., Hilpert, A., & Heethoff, M. (2017). Biomarker function and nutritional stoichiometry of neutral lipid fatty acids and amino acids in oribatid mites. Soil Biology & Biochemistry, 115, 35–43. 10.1016/j.soilbio.2017.07.020

[ece37894-bib-0007] Brückner, A., Schuster, R., Smit, T., Pollierer, M. M., Schäffler, I., & Heethoff, M. (2018). Track the snack – olfactory cues shape foraging behaviour of decomposing soil mites (Oribatida). Pedobiologia, 66, 74–80. 10.1016/j.pedobi.2017.10.004

[ece37894-bib-0008] Budge, S. M., Penney‐Belbin, S., & Lall, S. (2012). Estimating diets of Atlantic salmon (Salmo salar) using fatty acid signature analyses; validation with controlled feeding studies. Canadian Journal of Fisheries and Aquatic Sciences, 69, 1033–1046. 10.1139/f2012-039

[ece37894-bib-0009] Budge, S. M., Townsend, K., Lall, S. P., & Bromaghin, J. F. (2020). Dietary fat concentrations influence fatty acid assimilation patterns in Atlantic Pollock (*Pollachius* *virens*). Philosophical Transactions of the Royal Society B, 375, 20190649. 10.1098/rstb.2019.0649 PMC7333961 32536304

[ece37894-bib-0010] Buse, T., Ruess, L., & Filser, J. (2013). New trophic biomarkers for Collembola reared on algal diets. Pedobiologica, 56, 153–159. 10.1016/j.pedobi.2013.03.005

[ece37894-bib-0011] Chamberlain, P. M., Bull, I. D., Black, H. I. J., Ineson, P., & Evershed, R. P. (2005). Fatty acid composition and change in Collembola fed differing diets: Identification of trophic biomarkers. Soil Biology and Biochemistry, 37, 1608–1624. 10.1016/j.soilbio.2005.01.022

[ece37894-bib-0012] Chamberlain, P. M., Bull, I. D., Black, H. I. J., Ineson, P., & Evershed, R. P. (2006). Collembolan trophic preferences determined using fatty acid distributions and compound‐specific stable carbon isotope values. Soil Biology and Biochemistry, 38, 1275–1281. 10.1016/j.soilbio.2005.09.022

[ece37894-bib-0013] Endlweber, K., Ruess, L., & Scheu, S. (2009). Collembola switch diet in presence of plant roots thereby functioning as herbivores. Soil Biology and Biochemistry, 41, 1151–1154. 10.1016/j.soilbio.2009.02.022

[ece37894-bib-0014] Fernando‐Warnakulasuriya, G. J. P., Tsuchida, K., & Wells, M. A. (1988). Effect of dietary lipid content on lipid transport and storage during larval development of *Manduca* *sexta* . Insect Biochemistry, 18, 211–214. 10.1016/0020-1790(88)90025-X

[ece37894-bib-0015] Goetsch, C., Conners, M. G., Budge, S. M., Mitani, Y., Walker, W. A., Bromaghin, J. F., Simmons, S. E., Reichmuth, C., & Costa, D. P. (2018). Energy‐rich mesopelagic fishes revealed as a critical prey resource for a deep‐diving predator using quantitative fatty acid signature analysis. Frontiers in Marine Science, 5, 430. 10.3389/fmars.2018.00430

[ece37894-bib-0016] Happel, A., Stratton, L., Kolb, C., Hays, C., Richard, J., & Czesny, S. (2016). Evaluating quantitative fatty acid signature analysis (QFASA) in fish using controlled feeding experiments. Canadian Journal of Fisheries and Aquatic Sciences, 73, 1222–1229. 10.1139/cjfas-2015-0328

[ece37894-bib-0017] Haubert, D., Häggblom, M. M., Langel, R., Scheu, S., & Ruess, L. (2006). Trophic shift of stable isotopes and fatty acids in Collembola on bacterial diets. Soil Biology and Biochemistry, 38, 2004–2007. 10.1016/j.soilbio.2005.11.031

[ece37894-bib-0018] Heijboer, A., Ruess, L., Traugott, M., Jousset, A., & de Ruiter, P. (2017). Empirical methods of identifying and quantifying trophic interactions for constructing soil food web models. In J. Moore, P. de Ruiter, K. McCann, & V. Wolters (Eds.), Adaptive food webs: Stability and transitions of real and model ecosystems (pp. 257–286). Cambridge University Press.

[ece37894-bib-0019] Iverson, S. J., Field, C., Bowen, W. D., & Blanchard, W. (2004). Quantitative fatty acid analysis: A new method of estimating predator diets. Ecological Monographs, 74, 211–235. 10.1890/02-4105

[ece37894-bib-0020] Juárez, P., Chase, J., & Blomquist, G. J. (1992). A microsomal fatty acid synthetase from the integument of *Blattella* *germanica* synthesizes methyl‐branched fatty acids, precursors to hydrocarbon and contact sex pheromone. Archives of Biochemistry and Biophysics, 293, 333–341. 10.1016/0003-9861(92)90403-J 1536569

[ece37894-bib-0021] Kühn, J., Schweitzer, K., & Ruess, L. (2019). Diversity and specificity of lipid patterns in basal soil food web resources. PLoS One, 14, 1–16. 10.1371/journal.pone.0221102 PMC6701827 31430306

[ece37894-bib-0022] Kühn, J., Tobias, K., Jähngen, A., & Ruess, L. (2020). Shifting systems: Prerequisites for the application of quantitative fatty acid signature analysis in soil food webs. Philosophical Transactions of the Royal Society B, 375, 20190650. 10.1098/rstb.2019.0650 PMC7333970 32536311

[ece37894-bib-0023] Magnone, L., Bessonart, M., Rocamora, M., Gadea, J., & Salhi, M. (2015). Diet estimation of *Paralichthys* *orbignyanus* in a coastal lagoon via quantitative fatty acid signature analysis. Journal of Experimental Marine Biology and Ecology, 462, 36–49. 10.1016/j.jembe.2014.10.008

[ece37894-bib-0024] Malcicka, M., Ruther, J., & Ellers, J. (2017). De novo synthesis of linoleic acid in multiple Collembola Species. Journal of Chemical Ecology, 43, 911–919. 10.1007/s10886-017-0878-0 28823016

[ece37894-bib-0025] Menzel, R., Ngosong, C., & Ruess, L. (2017). Isotopologue profiling enables insights into dietary routing and metabolism of trophic biomarker fatty acids. Chemoecology, 27, 101–114. 10.1007/s00049-017-0236-2

[ece37894-bib-0026] Meyer, B. J., Mann, N. J., Lewis, J. L., Milligan, G. C., Sinclair, A. J., & Howe, P. R. (2003). Dietary intakes and food sources of omega‐6 and omega‐3 polyunsaturated fatty acids. Lipids, 38, 391–398. 10.1007/s11745-003-1074-0 12848284

[ece37894-bib-0027] Meynier, L., Morel, P. C. H., Chilvers, B. L., Mackenzie, D. D. S., & Duignan, P. J. (2010). Quantitative fatty acid signature analysis on New Zealand sea lions: Model sensitivity and diet estimates. Journal of Mammalogy, 9, 1484–1495. 10.1644/09-MAMM-A-299.1

[ece37894-bib-0028] Michaud, M. R., & Denlinger, D. L. (2006). Oleic acid is elevated in cell membranes during rapid cold‐hardening and pupal diapause in the flesh fly, *Sarcophaga* *crassipalpis* . Journal of Insect Physiology, 52, 1073–1082. 10.1016/j.jinsphys.2006.07.005 16997319

[ece37894-bib-0029] Ngosong, C., Gabriel, E., & Ruess, L. (2014). Collembola grazing on arbuscular mycorrhiza fungi modulates nutrient allocation in plants. Pedobiologia, 57, 171–179. 10.1016/j.pedobi.2014.03.002

[ece37894-bib-0030] Oksanen, J., Blanchet, F. G., Friendly, M., Kindt, R., Legendre, P., Mcglinn, D., Minchin, P. R., O'Hara, R. B., Simpson, G. L., Solymos, P., Stevens, M. H. M., Szoecs, E., & Wagner, H. (2018). vegan: community ecology package. R package version 2.5‐2. https://CRAN.Rproject.org/package=vegan

[ece37894-bib-0033] Ponge, J. F. (2000). Vertical distribution of Collembola (Hexapoda) and their food resources in organic horizons of beech forests. Biology and Fertility of Soils, 32, 508–522. 10.1007/s003740000285

[ece37894-bib-0034] Potapov, A. M., Tiunov, A. V., Scheu, S., Larsen, T., & Pollierer, M. M. (2019). Combining bulk and amino acid stable isotope analyses to quantify trophic level and basal resources of detritivores: A case study. Oecologia, 189, 447–460. 10.1007/s00442-018-04335-3 30659383

[ece37894-bib-0035] Rosen, D. A. S., & Tollit, D. J. (2012). Effects of phylogeny and prey type on fatty acid calibration coefficients in three pinniped species: Implications for the QFASA dietary quantification technique. Marine Ecology Progress Series, 467, 263–276. 10.3354/meps09934

[ece37894-bib-0036] Rosumek, F. B., Brückner, A., Blüthgen, N., Menzel, F., & Heethoff, M. (2017). Patterns and dynamics of neutral lipid fatty acids in ants – implications for ecological studies. Frontiers in Zoology, 14, 1–14. 10.1186/s12983-017-0221-1 28717381 PMC5508481

[ece37894-bib-0037] Ruess, L., & Chamberlain, P. M. (2010). The fat that matters: Soil food web analysis using fatty acids and their carbon stable isotope signature. Soil Biology and Biochemistry, 42, 1898–1910. 10.1016/j.soilbio.2010.07.020

[ece37894-bib-0038] Ruess, L., Schütz, K., Haubert, D., Häggblom, M. M., Kandeler, E., & Scheu, S. (2005). Application of lipid analysis to understand trophic interactions in soil. Ecology, 86, 2075–2082. 10.1890/04-1399

[ece37894-bib-0039] Scheu, S. (2002). The soil food web: Structure and perspectives. European Journal of Soil Biology, 38, 147–156. 10.1016/S1164-5563(01)01117-7

[ece37894-bib-0040] Stanley‐Samuelson, D. W., Jurenka, R. A., Cripps, C., Blomquist, G. J., & de Renobales, M. (1988). Fatty acids in insects: Composition, metabolism, and biological significance. Archives of Insect Biochemistry and Physiology, 9, 1–33. 10.1002/arch.940090102

[ece37894-bib-0041] Traugott, M., Kamenova, S., Ruess, L., Seeber, J., & Plantegenest, W. (2013). Characterising trophic networks: What emerging DNA based methods, stable isotopes and fatty acid analyses can offer. In G. Woodward, & D. A. Bohan (Eds.), Advances in ecological research: Ecological networks in an agricultural world (pp. 177–224). Academic Press.

[ece37894-bib-0042] Twining, C. W., Taipale, S. J., Ruess, L., Bec, A., Martin‐Creuzburg, D., & Kainz, M. J. (2020). Stable isotopes of fatty acids: Current and future perspectives for advancing trophic ecology. Philosophical Transactions of the Royal Society B: Biological Sciences, 375, 20190641. 10.1098/rstb.2019.0641 PMC7333957 32536315

[ece37894-bib-0043] Williams, C. T., & Buck, C. L. (2010). Using fatty acids as dietary tracers in seabird trophic ecology: Theory, applications and limitations. Journal of Ornithology, 151, 531–543. 10.1007/s10336-010-0513-0

[ece37894-bib-0044] Zelles, L. (1999). Fatty acid patterns of phospholipids and lipopolysaccharides in the characterisation of microbial communities in soil: A review. Biology and Fertility of Soils, 29, 111–129. 10.1007/s003740050533

