# Supplementary material for: Improving the application of quantitative fatty acid signature analysis in soil food webs: The effects of diet fat content
Source: Ecol Evol. 2021 Jul 9;11(16):11065–76. doi: 10.1002/ece3.7894 (PMC8366837; doi:10.1002/ece3.7894)
Supplement: Supplementary file 1 — Table S1 [file ECE3-11-11065-s002.docx]

**Table S1:** Overview the experimental treatments. Presented are the feeding trials conducted for the three Collembola consumers *Protaphorura fimata*, *Folsomia candida* and *Lepidocyrtus violaceus* in 3- and 6-day feeding experiments. Given is the name of the spiked fatty acid in the formulated diet and the proportion of added fatty acid.

| *P.fimata* | *F.candida* | *L.violaceus* |
| --- | --- | --- |
| 3 Days | | |
| yeast | yeast | yeast |
| 18:1ω9 – 5% | 18:1ω9 – 5% | 18:1ω9 – 5% |
| 18:1ω9 – 10% | 18:1ω9 – 10% | 18:1ω9 – 10% |
| 18:1ω9 – 15% | 18:1ω9 – 15% | 18:1ω9 – 15% |
| 18:2ω6,9 – 5% | 18:2ω6,9 – 5% | 18:2ω6,9 – 5% |
| 18:2ω6,9 – 10% | 18:2ω6,9 – 10% | 18:2ω6,9 – 10% |
| 18:2ω6,9 – 15% | 18:2ω6,9 – 15% | 18:2ω6,9 – 15% |
| a15:0 – 5% | a15:0 – 5% | a15:0 – 5% |
| a15:0 – 10% | a15:0 – 10% | a15:0 – 10% |
| a15:0 – 15% | a15:0 – 15% | a15:0 – 15% |
| cy19:0 – 5% | cy19:0 – 5% | cy19:0 – 5% |
| cy19:0 – 10% | cy19:0 – 10% | cy19:0 – 10% |
| cy19:0 – 15% | cy19:0 – 15% | cy19:0 – 15% |
| 16:1ω5 – 5% | 16:1ω5 – 5% | 16:1ω5 – 5% |
| 16:1ω5 – 10% | 16:1ω5 – 10% | 16:1ω5 – 10% |
| 16:1ω5 – 15% | 16:1ω5 – 15% | 16:1ω5 – 15% |
| 16:3ω3 – 5% | 16:3ω3,6,9 – 5% | - |
| 16:3ω3,6,9 – 10% | 16:3ω3,6,9 – 10% | - |
| 16:3ω3, 6,9 – 15% | 16:3ω3,6,9 – 15% | - |
| 18:1ω9 & a15:0 – 5% | 18:1ω9 & a15:0 – 5% | 18:1ω9 & a15:0 – 5% |
| 18:1ω9 & a15:0 & cy19:0 – 5% | 18:1ω9 & a15:0 & cy19:0 – 5% | 18:1ω9 & a15:0 & cy19:0 – 5% |
| 6 Days | | |
| yeast | yeast | yeast |
| 18:1ω9 – 5% | 18:1ω9 – 5% | 18:1ω9 – 5% |
| 18:1ω9 – 10% | 18:1ω9 – 10% | 18:1ω9 – 10% |
| 18:1ω9 – 15% | 18:1ω9 – 15% | 18:1ω9 – 15% |
| 18:2ω6,9 – 5% | 18:2ω6 – 5% | 18:2ω6 – 5% |
| 18:2ω6,9 – 10% | 18:2ω6 – 10% | 18:2ω6 – 10% |
| 18:2ω6,9 – 15% | 18:2ω6 – 15% | 18:2ω6 – 15% |
| a15:0 – 5% | a15:0 – 5% | a15:0 – 5% |
| a15:0 – 10% | a15:0 – 10% | a15:0 – 10% |
| a15:0 – 15% | a15:0 – 15% | a15:0 – 15% |
| cy19:0 – 5% | cy19:0 – 5% | cy19:0 – 5% |
| cy19:0 – 10% | cy19:0 – 10% | cy19:0 – 10% |
| cy19:0 – 15% | cy19:0 – 15% | cy19:0 – 15% |
| 16:1ω5 – 5% | 16:1ω5 – 5% | 16:1ω5 – 5% |
| 16:1ω5 – 10% | 16:1ω5 – 10% | 16:1ω5 – 10% |
| 16:1ω5 – 15% | 16:1ω5 – 15% | 16:1ω5 – 15% |
| 16:3ω3,6,9 – 5% | 16:3ω3,6,9 – 5% | 16:3ω3,6,9 – 5% |
| 16:3ω3,6,9 – 10% | 16:3ω3,6,9 – 10% | 16:3ω3,6,9 – 10% |
| 16:3ω3,6,9 – 15% | 16:3ω3,6,9 – 15% | 16:3ω3,6,9 – 15% |
