# Supplementary material for: Improving the application of quantitative fatty acid signature analysis in soil food webs: The effects of diet fat content
Source: Ecol Evol. 2021 Jul 9;11(16):11065–76. doi: 10.1002/ece3.7894 (PMC8366837; doi:10.1002/ece3.7894)
Supplement: Supplementary file 3 — Table S3 [file ECE3-11-11065-s001.docx]

**Table S3:** Diet estimation for the three Collembola consumers *Protaphorura fimata*, *Folsomia candida* and *Lepidocyrtus violaceus.* Comparison of results of a prior study to estimation of the same mixed diets with a new set of calibration coefficients calculated from the equations formulated from Figure 5. Presented are the mean estimated diet proportions [% ± s.d.] in the feeding trials after Kühn et al. 2020 (Old est.) as well as the proportions estimated using the new CC set presented in this study (New est.). Main diet components are marked in bold.

| **Diet** | ***F. candida*** | | ***P. fimata*** | | ***L. violaceus*** | |
| --- | --- | --- | --- | --- | --- | --- |
|  | Old est. | New est. | Old est. | New est. | Old est. | New est. |
| **Algae – Bacteria 50:50** | | | | | | |
| **Algae** | **50.5** ± 2.5 | **39.6** ± 3.5 | **38.8** ± 3.1 | **51.0** ± 3.5 | **37.7** ± 2.3 | **58.8** ± 3.4 |
| **Bacteria** | **18.4** ± 0.0 | **20.9** ± 3.6 | **10.6** ± 0.0 | **8.2** ± 1.6 | **12.9** ± 0.0 | **12.4** ± 1.9 |
| Fungi | 0.1 ± 0.2 | 39.5 ± 2.2 | 0.0 ± 0.0 | 6.6 ± 5.7 | 0.0 ± 0.0 | 0.0 ± 0.0 |
| Leaves | 0.0 ± 0.0 | 0.0 ± 0.0 | 13.9 ± 0.3 | 8.9 ± 0.4 | 25.4 ± 2.5 | 19.9 ± 2.7 |
| Roots | 31.0 ± 3.3 | 0.0 ± 0.0 | 36.6 ± 3.7 | 25.3 ± 2.1 | 24.0 ± 2.4 | 8.9 ± 3.6 |
| **Algae – Fungi 50:50** | | | | | | |
| **Algae** | **11.7** ± 0.2 | **45.3** ± 1.0 | **15.6** ± 0.7 | **20.9** ± 0.5 | **25.0** ± 1.4 | **45.2** ± 3.7 |
| Bacteria | 6.6 ± 0.0 | 8.5 ± 1.2 | 1.9 ± 0.0 | 1.6 ± 0.1 | 6.6 ± 0.0 | 6.5 ± 0.8 |
| **Fungi** | **26.9** ± 1.5 | **46.2** ± 1.6 | **27.4** ± 3.8 | **39.8** ± 2.3 | **0.0** ± 0.0 | **10.3** ± 6.0 |
| Leaves | 0.0 ± 0.0 | 0.0 ± 0.0 | 16.2 ± 2.2 | 14.2 ± 1.9 | 19.4 ± 1.4 | 19.2 ± 1.3 |
| Roots | 54.8 ± 0.5 | 0.0 ± 0.0 | 38.9 ± 1.2 | 23.6 ± 0.5 | 49.0 ± 0.0 | 18.8 ± 1.8 |
| **Algae – Leaves 50:50** | | | | | | |
| **Algae** | **14.6** ± 9.4 | **54.8** ± 39.9 | **40.2** ± 1.4 | **56.2** ± 2.4 | **13.9** ± 7.4 | **21.8** ± 13.6 |
| Bacteria | 6.3 ± 0.0 | 5.2 ± 0.4 | 1.4 ± 0.0 | 1.2 ± 0.2 | 6.0 ± 0.0 | 5.8 ± 0.4 |
| Fungi | 26.1 ± 45.2 | 38.4 ± 42.3 | 0.0 ± 0.0 | 1.5 ± 2.5 | 0.0 ± 0.0 | 36.3 ± 18.1 |
| **Leaves** | **3.9** ± 3.4 | **0.0** ± 0.0 | **27.6** ± 2.5 | **18.8** ± 2.5 | **21.2** ± 4.2 | **24.5** ± 6.8 |
| Roots | 49.1 ± 31.0 | 2.7 ± 2.4 | 30.8 ± 3.0 | 22.2 ± 1.7 | 58.9 ± 6.7 | 11.6 ± 7.2 |
| **Algae – Roots 50:50** | | | | | | |
| **Algae** | **63.4** ± 1.0 | **59.4** ± 7.4 | **49.5** ± 3.7 | **62.0** ± 4.4 | **18.6** ± 8.6 | **30.1** ± 13.8 |
| Bacteria | 7.4 ± 0.0 | 7.5 ± 2.5 | 1.7 ± 0.0 | 1.4 ± 0.2 | 5.2 ± 0.0 | 4.6 ± 0.8 |
| Fungi | 0.0 ± 0.0 | 33.0 ± 5.7 | 7.1 ± 2.4 | 6.6 ± 3.1 | 0.0 ± 0.0 | 3.6 ± 6.1 |
| Leaves | 0.0 ± 0.0 | 0.0 ± 0.0 | 0.7 ± 0.2 | 0.0 ± 0.0 | 36.9 ± 2.6 | 37.8 ± 4.1 |
| **Roots** | **29.3** ± 1.6 | **0.0** ± 0.0 | **41.0** ± 3.3 | **29.6** ± 2.7 | **39.4** ± 8.0 | **23.9** ± 4.2 |
| **Fungi – Bacteria 50:50** | | | | | | |
| Algae | 1.1 ± 0.0 | 1.1 ± 0.0 | 2.04 ± 0.0 | 4.6 ± 0.0 | 6.1 ± 0.1 | 11.6 ± 0.2 |
| **Bacteria** | **17.0** ± 0.0 | **19.9** ± 1.4 | **19.08** ± 0.0 | **15.6** ± 1.1 | **16.0** ± 0.0 | **16.6** ± 0.0 |
| **Fungi** | **38.0** ± 3.4 | **75.3** ± 1.2 | **22.48** ± 2.7 | **47.9** ± 1.2 | **0.0** ± 0.0 | **51.3** ± 3.0 |
| Leaves | 0.0 ± 0.0 | 3.7 ± 0.4 | 1.33 ± 0.3 | 0.9 ± 0.7 | 14.8 ± 1.3 | 20.5 ±2.3 |
| Roots | 43.9 ± 5.0 | 0.0 ± 0.0 | 55.08 ± 1.2 | 31.0 ± 0.3 | 63.1 ± 1.3 | 0.4 ± 0.7 |
| **Fungi – Leaves 50:50** | | | | | | |
| Algae | 0.7 ± 0.6 | 0.2 ± 0.2 | 3.6 ± 0.6 | 9.8 ± 1.5 | 4.9 ± 0.3 | 8.4 ± 0.5 |
| Bacteria | 4.7 ± 0.0 | 4.9 ± 2.0 | 1.8 ± 0.0 | 1.8 ± 0.1 | 5.8 ± 0.0 | 5.7 ± 0.1 |
| **Fungi** | **53.8** ± 3.8 | **70.6** ± 3.9 | **51.6** ± 3.4 | **52.3** ± 1.8 | **0.0** ± 0.0 | **45.0** ± 5.4 |
| **Leaves** | **22.7** ± 7.1 | **24.3** ± 5.6 | **9.7** ± 0.5 | **9.0** ± 1.2 | **18.6** ± 1.6 | **28.1** ± 3.1 |
| Roots | 18.1 ± 8.6 | 0.0 ± 0.0 | 33.2 ± 3.9 | 27.1 ± 2.4 | 70.7 ± 1.3 | 12.7 ± 3.6 |
| **Fungi – Roots 50:50** | | | | | | |
| Algae | 0.7 ± 0.1 | 0.4 ± 0.1 | 5.41 ± 0.5 | 11.6 ± 0.9 | 5.3 ± 2.2 | 8.9 ± 2.7 |
| Bacteria | 3.7 ± 0.0 | 3.2 ± 0.6 | 1.76 ± 0.0 | 1.9 ± 0.1 | 6.4 ± 0.0 | 6.6 ± 0.7 |
| **Fungi** | **72.8** ± 11.7 | **92.5** ± 2.6 | **65.32** ± 0.9 | **62.3** ± 0.1 | **31.0** ± 53.7 | **75.0** ± 12.4 |
| Leaves | 0.0 ± 0.0 | 3.8 ± 0.0 | 1.76 ± 0.1 | 0.0 ± 0.0 | 7.9 ± 6.9 | 7.6 ± 6.8 |
| **Roots** | **22.8** ± 11.2 | **0.0** ± 0.0 | **25.76** ± 1.3 | **24.2** ± 0.7 | **49.3** ± 42.8 | **1.8** ± 3.2 |
| **Leaves – Roots 50:50** | | | | | | |
| Algae | 2.6 ± 0.1 | 1.90 ± 0.3 | 1.58 ± 0.1 | 3.6 ± 0.1 | 2.0 ± 0.1 | 0.3 ± 0.1 |
| Bacteria | 14.8 ± 0.0 | 11.60 ± 1.8 | 2.76 ± 0.0 | 2.2 ± 0.1 | 6.3 ± 0.0 | 4.9 ± 0.0 |
| Fungi | 22.5 ± 5.6 | 57.80 ± 3.3 | 30.82 ± 2.7 | 39.3 ± 3.8 | 0.0 ± 0.0 | 22.4 ± 3.5 |
| **Leaves** | **5.2** ± 0.0 | **28.70** ± 4.7 | **7.68** ± 0.5 | **14.5** ± 0.5 | **60.0** ± 1.4 | **63.5** ± 1.7 |
| **Roots** | **55.0** ± 3.9 | **0.00** ± 0.0 | **57.16** ± 2.7 | **40.4** ± 0.0 | **31.7** ± 1.4 | **6.8** ± 1.8 |
| **Algae – Bacteria – Fungi 33:33:33** | | | | | | |
| **Algae** | **7.8** ± 0.5 | **29.1** ± 3.6 | **15.59** ± 1.1 | **23.6** ± 0.1 | **14.2** ± 8.3 | **23.9** ± 1.4 |
| **Bacteria** | **10.7** ± 0.0 | **11.6** ± 1.0 | **11.25** ± 0.0 | **9.7** ± 0.0 | **12.0** ± 0.0 | **11.9** ± 0.8 |
| **Fungi** | **26.7** ± 5.3 | **59.3** ± 4.6 | **10.01** ± 2.7 | **32.2** ± 2.5 | **0.0** ± 0.0 | **29.0** ± 11.9 |
| Leaves | 0.0 ± 0.0 | 0.0 ± 0.0 | 2.06 ± 0.5 | 0.0 ± 0.0 | 16.9 ± 4.3 | 20.4 ± 11.2 |
| Roots | 54.8 ± 0.9 | 0.0 ± 00 | 61.10 ± 2.7 | 35.2 ± 2.6 | 57.0 ± 4.4 | 14.8 ± 11.1 |
| **Algae –Fungi – Leaves 33:33:33** | | | | | | |
| **Algae** | **49.1** ± 3.3 | **52.5** ± 3.9 | **35.1** ± 1.5 | **45.3** ± 1.9 | **21.0** ± 0.8 | **34.6** ± 2.0 |
| Bacteria | 7.2 ± 0.0 | 7.0 ± 1.7 | 1.7 ± 0.0 | 1.5 ± 0.1 | 6.2 ± 0.0 | 6.1 ± 0.1 |
| **Fungi** | **0.0** ± 0.0 | **40.6** ± 2.6 | **2.4** ± 2.1 | **15.5** ± 1.9 | **0.0** ± 0.0 | **26.8** ± 5.8 |
| **Leaves** | **1.5** ± 1.3 | **0.0** ± 0.0 | **24.2** ± 2.8 | **14.4** ± 2.0 | **20.8** ± 1.9 | **20.6** ± 2.1 |
| Roots | 42.2 ± 3.4 | 0.0 ± 0.0 | 36.6 ± 1.5 | 23.3 ± 0.8 | 52.2 ± 2.1 | 11.9 ± 2.4 |
